# Patient Experience and Expectations in Oral Health Care: A Nation-Wide Survey

**DOI:** 10.1016/j.identj.2024.10.011

**Published:** 2024-11-06

**Authors:** Liran Levin, Anahat Khehra, Sharon Kowal, Karla Romer

**Affiliations:** a College of Dentistry, University of Saskatchewan, Saskatoon, Saskatchewan, Canada; b Division of Periodontology, Department of Oral Medicine, Infection and Immunity, Harvard School of Dental Medicine, Boston, Massachusetts, USA; c Analytics & Insights, Procter & Gamble, Cincinnati, Ohio, USA

**Keywords:** Prevention, Oral hygiene, Patient motivation, Dental care, Plaque, Patient-centred care

## Abstract

**Aim:**

This research was conducted to understand patients’ dental visit habits and behaviours and to identify factors contributing to a positive patient experience at the dental office.

**Methods:**

An online survey was distributed to members of a market research panel in the United States. Qualifying panellists were 25 years of age or older who visited a dentist in the past year. Survey questions were related to frequency of dental visits, patient satisfaction with the dental office, importance of dental office attributes and dental product recommendation expectations.

**Results:**

There were 400 respondents, all from USA, with a mean age of 51 years; 54% were female and 46% were male. Overall, 74% had a college or post-graduate degree. The average number of dental visits per patient per year was 2.1. Fifty percent had been going to the same practice for more than 2 years. 84% of panellists indicated satisfaction with their dental office, which was driven by attributes related to good customer service and quality dental care/services. Factors rated as contributing to patient trust included: offering good services; polite and friendly behaviour; affordable cost; and clear and honest communication. Attributes rated as being most important for a dental practice included: valuing their time; not seeing them as just a patient; gentle dental team; and improving their oral health. Overall, 55% of respondents indicated they expect recommendations for specific brands to treat specific oral care issues.

**Conclusion:**

Patients are seeking a more personal connection with their dental office and are interested in receiving information about their oral care habits. Including personalised self-care recommendations as part of every dental treatment plan will address these needs and motivate patients to engage in their oral health care. Education, communication and building personal connections are keys to establish a positive patient experience.

## Introduction

The demands and expectations on oral health care providers are increasing.^1^ Patients are seeking high-quality care and personalised attention and education at an affordable cost. Moreover, there is a shift in focus on oral health-related quality of life, which is evidenced by a greater number of patients seeking answers regarding their oral health online.^2^ A wave of technological innovations and advancements have supported patient engagement outside of the health care office.^3^ For example, applications are available to coach and track behaviour on brushing habits.^4^ These factors contribute to an increase in patient expectations on their dental providers to provide optimal care.

To address health system performance, the Triple-Aim was introduced by Berwick and colleagues.^5^ It focuses on improving patient experience, improving health of populations and reducing per capita costs of care. Later, Bodenheimer and Sinky suggested the addition of a fourth aim: improving provider experience.^6^ It is recognised that an increase in patient demand is associated with a rise in health care provider burnout,^7^^,^^8^ therefore having the appropriate resources and support available for providers should facilitate the delivery of high-quality care. An increase in demand may put strain on time and attention health care professionals give to oral hygiene instruction, self-care education and preventive measures. Haverfield et al. conducted a systematic review to assess how patient-provider interpersonal interventions influence Quadruple-Aim outcomes.^9^ Interventions investigated included, among others, motivational interviewing, health literacy, communication and shared decision-making. The review found that minimal to moderate demands on provider time and effort may improve patient experience, provider experience and overall patient health. To ensure the Quadruple-Aim model is being met and to identify where interventions are needed, it is important to receive feedback from patients and providers.

The measurement of patient experience in medical care is common practice, however it is not applied as frequently in oral health care.^10^, ^11^, ^12^ It is important to measure patient experience as it provides information on what the patient needs and values.^10^, ^11^, ^12^ A patient-centric approach can be formulated and applied to improve communication, concordance and treatment outcomes. Karimbux et al. investigated the challenges in measuring patient experience of oral health care.^13^ Currently, no standardised measurement tool exists, and widespread use is hurdled by variations in care models across dental offices (solo practice, multi-speciality, dental service organizations), which could impact data interpretation, as well as lack of incentives for implementation. Despite this, ample opportunities are available to develop a survey tool that can be used to measure patient experiences in oral health care.

The present study was undertaken to understand patients’ dental visit habits and behaviours and to identify factors contributing to a positive patient experience at the dental office.

## Methods

An online survey was distributed to members of a market research panel in the United States by Ipsos, USA. Ipsos adheres to all local legislation and market research Industry rules and codes of conduct (e.g., Insights Association Code of Standards and Ethics). This market research panel is a third-party private tool that can be used to conduct and deliver surveys. The panel of interest contains 774, 206 potential respondents that are considered representative of the United States population. The potential respondents are validated and monitored for survey-taking behaviour to ensure thoughtful and engaged responses.

Panellists were pre-screened to identify those who were 25 years of age or older and had visited a dentist in the past year. Pre-screened panellists were invited to participate by email, ensuring good representation across demographic characteristics, until 400 responses were recorded. Prior to participation, study background information was provided and consent obtained. In addition, demographic information (age, gender, region, race, education) was recorded. The remaining questions were related to frequency of dental visits, patient satisfaction with the dental office, importance of dental office attributes and dental product recommendation expectations. A mix of multiple choice, short answer and numerical ranking questions was included (Appendix A).

The recorded responses were reviewed to ensure no duplications from the same participant. Statistical analysis was performed using SPSS Quanvert software. Coding of open-ended responses was done manually.

## Results

### Demographics

The survey received 400 responses, and all were from the United States. The mean age (SD) was 51 (15.5) years. Twenty-two percent of respondents were 25-34 years of age, 22% were 65 years or older and the remainder were evenly distributed (19% each) across the 35-44, 45-54 and 55-64 age categories. 54% were female and 46% were male. Overall, 74% had a college or post-graduate degree. Thirty-nine percent were from the South, 21% from the Midwest, 21% from the West and 19% from the Northeast. Sixty-six percent were White/Caucasian, 12% Hispanic/Latino, 12% Black/African-American, 7% Asian/Pacific Islander and 3% other. The average number of dental visits (SD) per patient per year was 2.1 (0.9). Five percent of respondents typically have less than one dental visit per year, 10% have one visit, 68% have two visits, 9% have 3 visits, 7% have four visits and 2% have five or more dental visits per year. Fifty percent had been going to the same practice for more than 2 years.

### Experience with current dental office

**Fig. 1:**
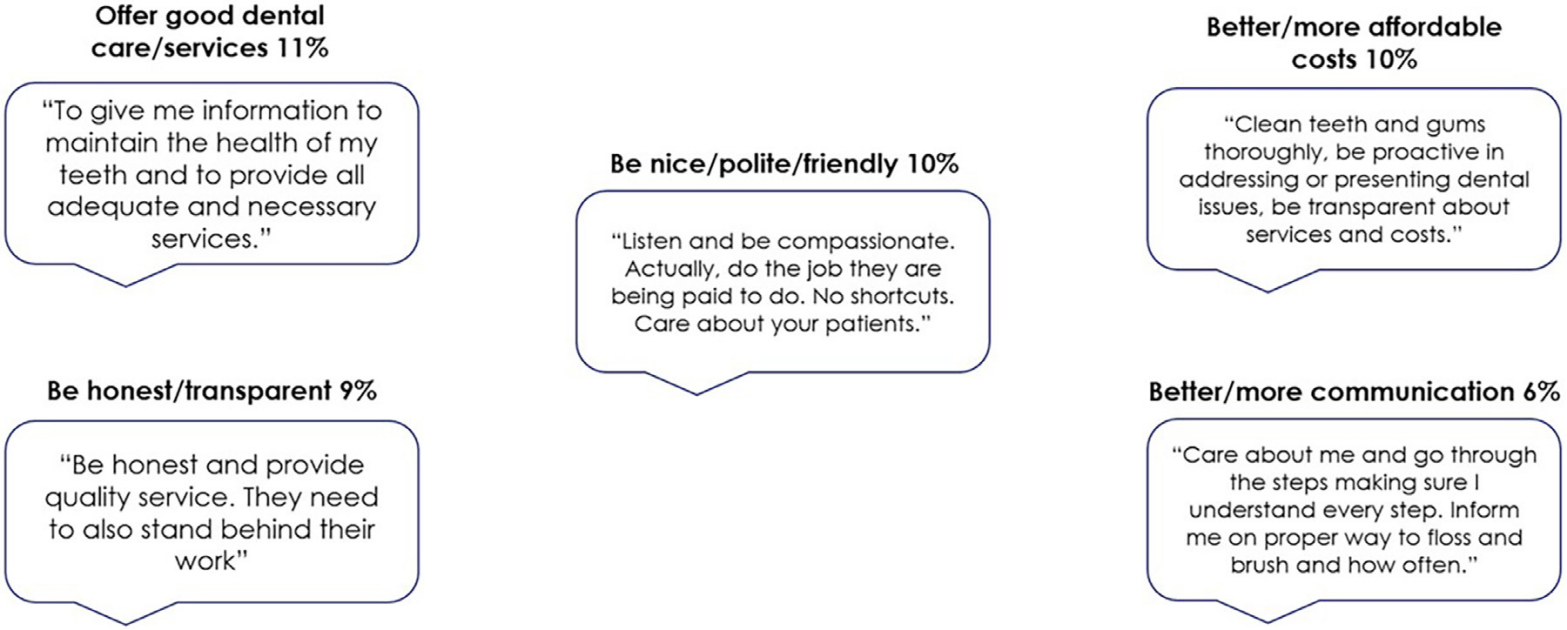
Top 5 patient's suggestions to gain trust (Appendix, Question 5).

**Fig. 2:**
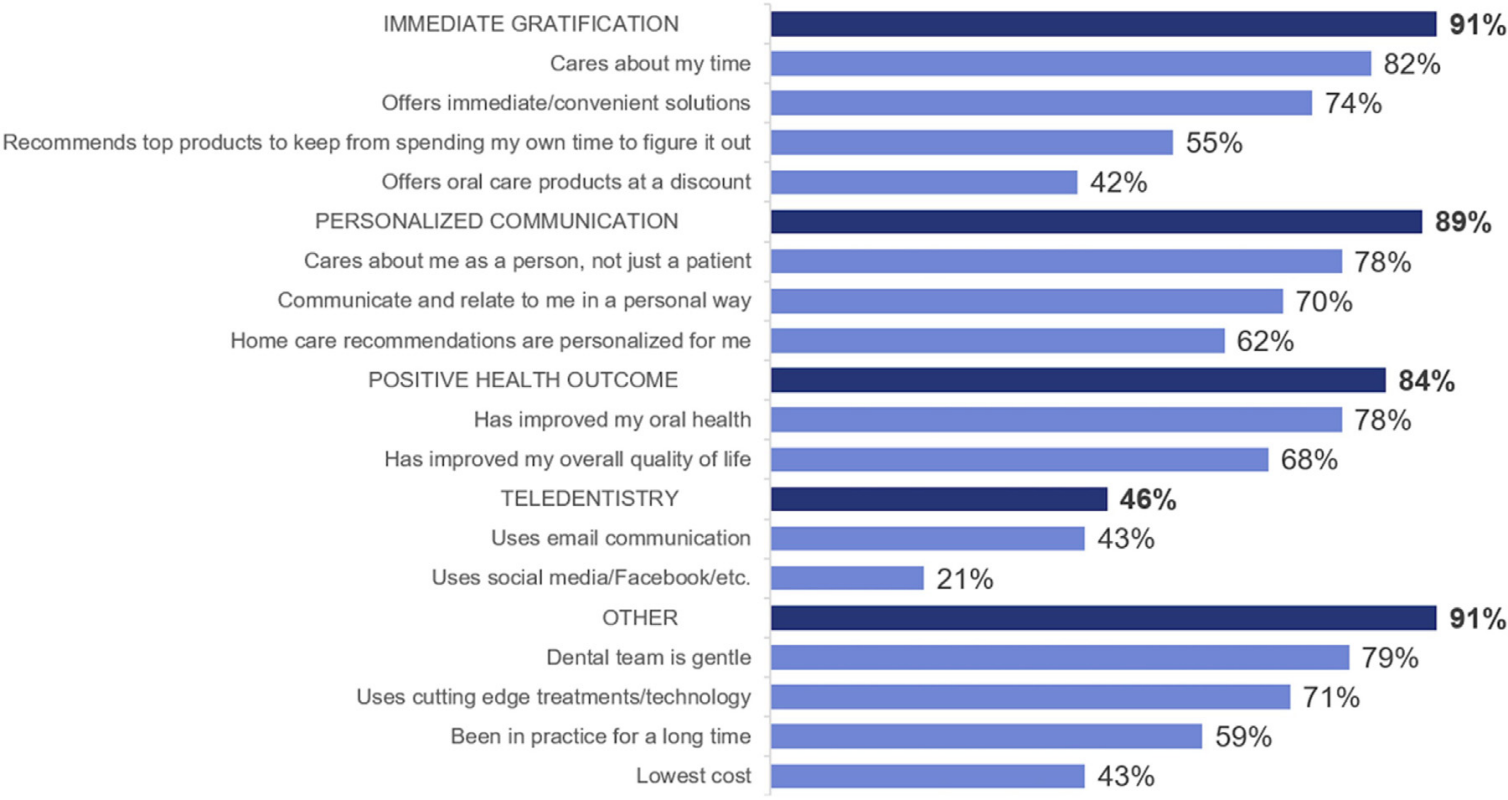
Patient importance rating of dental office attributes. The percentage next to purple bars indicates participants who chose 'extremely important' or 'very important' for the attribute. Headings with dark blue bars are descriptors for the attributes in each category. The headings were not shown to participants. The percentage of participants who chose 'extremely important' for at least one attribute in the category is indicated by the dark blue bars (Appendix, Question 6).

Overall, 84% of panellists indicated satisfaction with their dental office, 14% were somewhat satisfied and 2% were not satisfied. Attributes mentioned by respondents as driving satisfaction were related to good customer service (48%) and quality dental care/services (42%). Most common factors cited by respondents as contributing to patient trust included offering good services (11%); polite and friendly behaviour (10%); affordable cost (10%); transparency (9%) and clear communication (6%). Figure 1 outlines these suggestions alongside selected frequent patient quotes. The most important rated dental office attribute was 'cares about my time' (82% rated it extremely or very important), followed by 'dental team is gentle' (79%), 'cares about me as a person, not just a patient' (78%) and 'has improved my oral health (78%) (Figure 2). Furthermore, attributes related to immediate gratification, personalised communication and positive health outcomes have high importance to patients who are extremely likely to recommend the dental office, adhere to treatment recommendations, keep appointments and maintain a relationship with the dental office (Table 1).

**Table 1:** Importance of dental office attributes for respondents who said they would definitely recommend their dental office, follow treatment recommendations, keep appointments and/or maintain a relationship with their dental office.

	Ratings for respondents who said they would DEFINITELY….			
Dental office attributes	Refer practice 'Recommended dental office family & friends'	Follow treatment 'Follow treatment recommendation'	Keep appointment 'Keep my appointment'	Be loyal 'Keep a relationship with dental office'
Immediate gratification*	**97%**	**97%**	**96%**	**97%**
Cares about my time	93%	90%	90%	91%
Offers immediate/convenient solutions	86%	82%	84%	85%
Recommends top products to keep from spending my own time to figure it out	63%	65%	58%	62%
Offers oral care products at a discount	44%	44%	40%	44%
Personalised communication*	**96%**	**95%**	**95%**	**96%**
Cares about me as a person, not just a patient	91%	88%	88%	91%
Communicate and related to me in a personal way	82%	79%	77%	82%
Home care recommendations are personalised for me	67%	70%	66%	71%
Positive health outcome*	**94%**	**94%**	**91%**	**95%**
Has improved my oral health	89%	91%	87%	91%
Has improved my overall quality of life	81%	80%	76%	81%
Teledentistry*	**50%**	**50%**	**48%**	**51%**
Uses email communication	49%	48%	47%	50%
Uses social media/Facebook/etc.	21%	22%	20%	22%
Other*	**96%**	**97%**	**94%**	**96%**
Dental team is gentle	87%	88%	85%	88%
Uses cutting edge treatments/technology	79%	79%	78%	79%
Been in practice for a long time	69%	68%	65%	71%
Lowest cost	41%	42%	40%	44%

The percentage for attribute rows (those without an *) indicates the percentage of participants who chose “extremely important” or “very important” for the attribute (Appendix, Question 6).

⁎ Headings in the rows with an * are descriptors for the attributes listed in the category. The headings were not shown to participants. The percentages for each heading indicate the percentage of participants who chose “extremely important” for at least one attribute in the category.

### Expectations for self-care recommendations

**Fig. 3:**
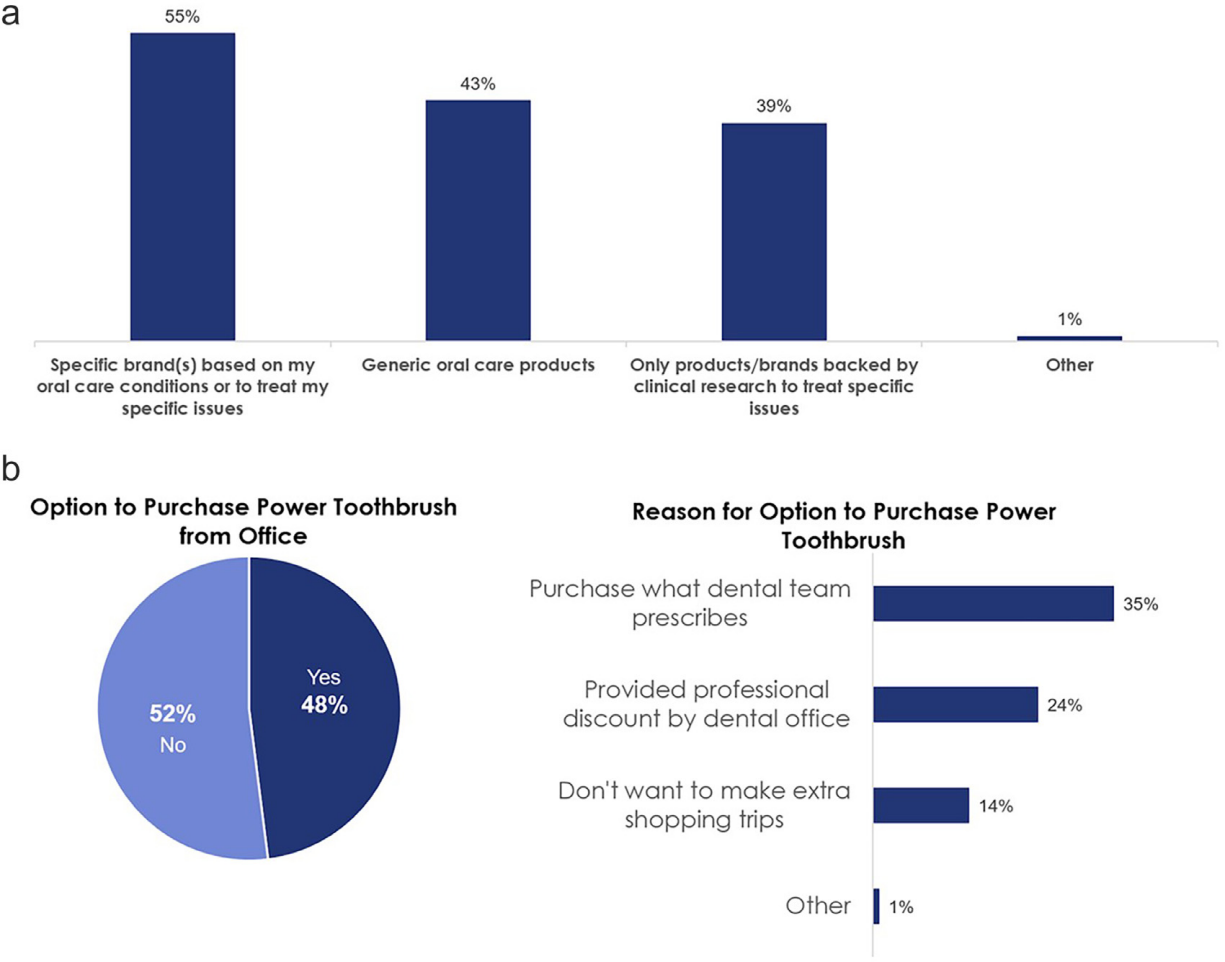
(**A)** Patient expectations for dental recommendations. (B) Desired purchasing options of recommended power toothbrushes (Appendix, Questions 8, 9 and 9a).

**Fig. 4:**
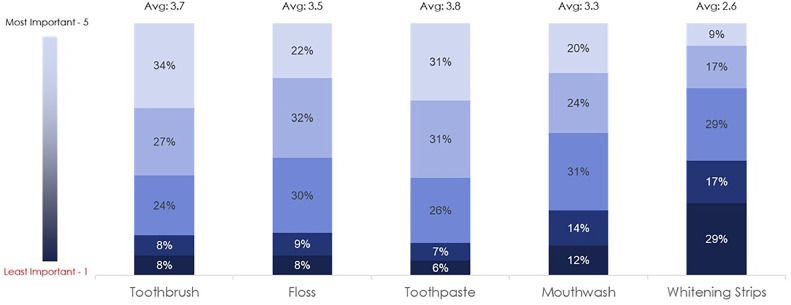
Ranked importance of dental office samples, including toothbrush, floss, toothpaste, mouthwash and whitening strips (Appendix, Question 10).

**Fig. 5:**
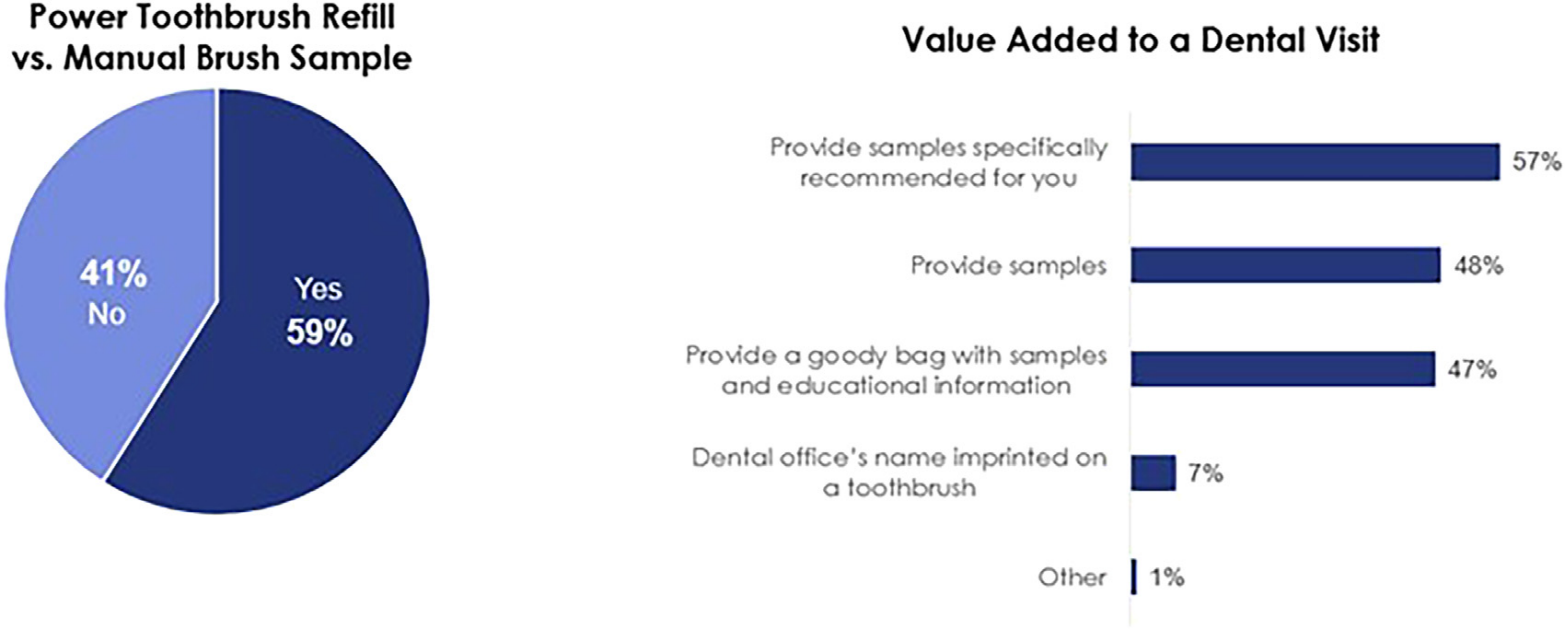
Preference and value perception of samples (Appendix, Questions 11 and 12).

The majority of respondents (55%) indicated they expect recommendations for specific brands to treat their specific oral care issues (Figure 3A). Figure 3B demonstrates that around half (48%) of the respondents would like the option to purchase a power toothbrush from their dental office. Accessibility to the product recommended was of high importance to the respondents. In addition, toothpaste and toothbrush samples were rated as the most important samples to respondents followed by floss, mouthwash and whitening strips (Figure 4). Fifty-nine percent of respondents would prefer to receive a power toothbrush refill instead of a manual toothbrush. Finally, approximately half of respondents said samples and educational material add value to the dental visit, with the largest percentage (57%) choosing ‘samples specifically recommended for me’ (Figure 5).

## Discussion

**Fig. 6:**
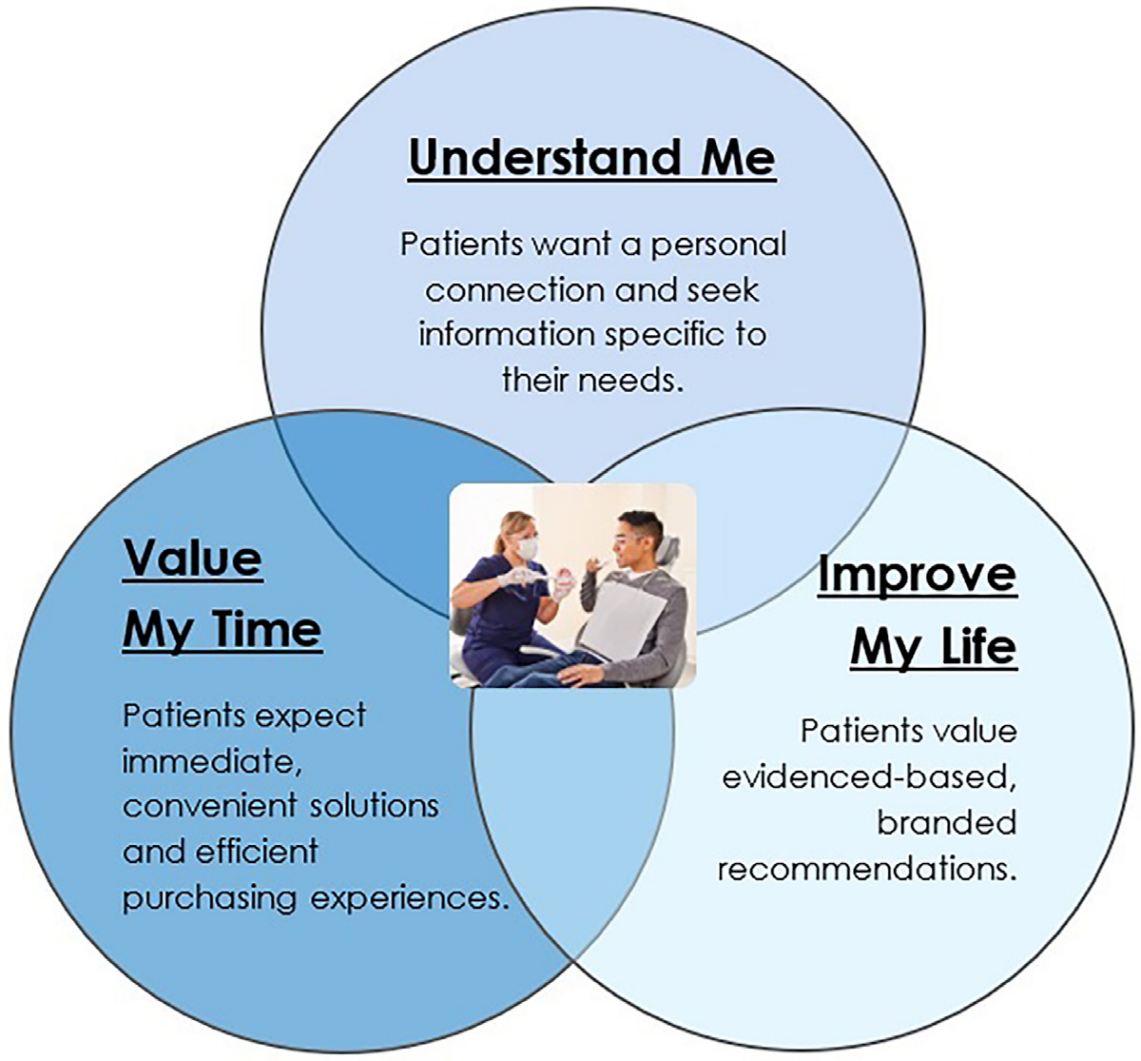
Key drivers of dental patient motivation.

An increased patient demand for high-quality and personalised oral health care calls for investigation of the patient experience. This nation-wide survey found that most respondents receive biannual preventive dental care and are satisfied with their dental office. The survey findings can be practically applied and related to 3 key drivers of dental patient motivation: understand me (attributes related to personalised communication), value my time (attributes related to immediate gratification) and improve my life (attributes related to positive health outcomes) (Figure 6).^14^

Patients want to be understood by their dental office and build a personal connection based on trust.^15^ A patient-tailored treatment plan will provide a more personalised approach to oral health care. In combination with effective provider communication, studies from the medical literature show personalization can lead to increased patient concordance, acceptance of recommendations and loyalty to the office.^7^^,^^14^^,^^16^, ^17^ Moreover, a dental office implementing patient-centred care may help patients take initiative to participate in their own oral health care, which is a fundamental aspect of patient care models.^18^^,^^19^ Patients who are self-motivated may achieve positive health outcomes, as seen in research involving medical patients.^20^^,^^21^ Sbaraini et al. conducted a similar study on the dental patient experience and found that respondents were more motivated to utilize oral hygiene products when being 'treated as a person and not as a patient.'^22^ The use of technology can also be implemented by the provider to continue connecting with and motivating patients between appointments.^23^

A second key driver of patient motivation is valuing the patient's time. In the survey, respondents were asked to rate dental professional attributes they thought were most important. Among the highest rated attributes included 'caring about my time' and 'offering immediate/convenient solutions.' The provider should maximize the value of time spent in each appointment by educating and coaching patients on how to improve their oral health status. Technology also offers numerous opportunities to educate and communicate with patients in a timely and efficient manner.^23^ About half of respondents indicated they would like to purchase power toothbrushes in the office, so dental teams could consider offering their most recommended oral hygiene products in the practice. In other studies, about half of the patients indicated they would prefer to purchase products directly from their dental office as it is immediate, convenient and efficient.^14^^,^^20^^,^^21^^,^^24^

Finally, improving the patient's life is another key factor driving patient motivation. 'Improving my oral health' was one of the most important attributes for a dental office in this study. The average patient interest in oral health has increased and detailed information on home care instruction is valued. Dedicating ample time to oral hygiene instruction and oral health education to prevent oral disease is consistent with recent guidance from the World Health Organization, which calls on oral health professionals to support patients in effective self-care behaviors.^25^ This may be accompanied by branded, evidence-based, self-care recommendations targeted to improve patients’ specific oral health conditions.^24^ Overall, 55% of survey respondents indicated they expect recommendations for specific brands.

Since most oral diseases originate from suboptimal plaque biofilm control, recommending preventive, evidence-based, self-care products as part of a personalised treatment plan is critical. Numerous systematic reviews and population-based research show electric toothbrushes provide significantly greater gingival health and plaque removal benefits compared to manual toothbrushes.^26^^,^^27^ Within the electric toothbrush category, oscillating-rotating toothbrushes have shown greater and faster gingival health improvements and greater plaque removal compared to sonic toothbrushes.^26^, ^27^, ^28^, ^29^ Oscillating-rotating toothbrushes are also preferred by more users over sonic toothbrushes,^29^ and they offer a personalised brushing experience. Patients can choose from a variety of brush heads and brushing modes, and interactive models provide individualised coaching via an app to improve brushing behaviours. Another core component of preventive self-care is fluoride dentifrice. Stannous fluoride (SnF_2_), an antibacterial fluoride, is the only fluoride clinically proven to significantly inhibit plaque regrowth, reduce plaque virulence and improve gingival health.^30^^,^^31^ Biesbrock et al. reported 3.7 times greater odds of transitioning a patient from gingivitis to periodontal health with the use of bioavailable gluconate chelated SnF_2_ toothpaste compared to a negative control and 2.8 times greater odds compared to a positive control.^31^ A combination of products may be considered in treatment planning based on each patient's needs.

In this study, the use of an experienced company to place the patient survey with its established panel of respondents ensured real and engaged responses. The panel consisted of a large base size that reflected the US population in terms of age, gender, race and geographic distribution.^32^, ^33^, ^34^ However, the mean education level and income were above average.^31^ As a result, the patient needs and values may be different and should be assessed in each socioeconomic group. Another limitation of the current study is the fact that the results are based on self-reporting which may have led to underreporting or exaggeration. Future research should validate the patient experience survey, which was developed for this learning study, with the aim to incorporate its use in various dental offices. It is important for dental professionals to measure the patient experience to improve delivery of care through personalised recommendations. A survey of the provider experience would also offer valuable insights to improve the patient-provider experience.

## Conclusions

Patients are seeking a more personal connection with their dental office and are interested in receiving information about their oral care habits. Patients would like to be understood, have their time valued and provided with brand-specific recommendations to improve their health. Including personalised self-care recommendations as part of every dental treatment plan will address these needs and motivate patients to engage in their oral health care. Future studies may further refine the patient experience survey and promote its widespread use.

## Declaration of competing interest

Liran Levin has done consulting work for Procter & Gamble. Anahat Khehra has no conflicts to disclose. Sharon Kowal is an employee of The Procter & Gamble Company. Karla Romer is a retired employee of The Procter & Gamble Company.

## Appendix Supplementary materials

Image, application 1

## Footnotes

Supplementary material associated with this article can be found in the online version at doi:10.1016/j.identj.2024.10.011.
