# Supplementary material for: Patient Experience and Expectations in Oral Health Care: A Nation-Wide Survey
Source: Int Dent J. 2024 Nov 6;75(2):1003–10. doi: 10.1016/j.identj.2024.10.011 (PMC11976609; doi:10.1016/j.identj.2024.10.011)
Supplement: Supplementary file 1 [file mmc1.docx]

**Appendix.**

**Patient Dental Experience Survey**

| **Dental Visit Habits** |
| --- |

**Q1. Frequency of Dental Visits**

In a typical year, how many times do you, yourself, typically visit your dental office for routine checkups?

(Select one)

1. Less than once per year
2. Once a year
3. 2 times per year
4. 3 times per year
5. 4 times per year
6. 5 times per year
7. 6 or more times per year

**Q2. Current Dental Office Duration**

How long have you been with your current dental office?

(Select one)

1. Within 1 month or less
2. Over a month but within the last 3 months
3. Over 3 months but within the last 6 months
4. Over 6 months but within the last 9 months
5. Over 9 months but within the last year
6. Over 1 year but within the last 2 years
7. Over 2 years
8. I have always been with this dental office

**Q3. Motivations to Increase Visit Frequency**

Would any of the following influence you to increase your visit frequency?

(Select all that apply)

1. My dental office educates me on how to optimize my oral health at home
2. My dental office offers an easy way for me to purchase and pick up their recommended home care products at a professional discount from the office
3. Other (specify)
4. Nothing

| **Patient Satisfaction** |
| --- |

**Q4. Overall Satisfaction**

Overall, how satisfied are you with your current dental office?

(Select one)

1. Extremely Satisfied
2. Very Satisfied
3. Somewhat Satisfied
4. Not Very Satisfied
5. Not at all Satisfied

**Q4a. Reasons for Satisfaction**

Why are you satisfied with your current dental office?

(Please be as specific as possible)

**Q4b. Reasons for Dissatisfaction**

Why are you not satisfied with your current dental office?

(Please be as specific as possible)

**Q5. Opinions on Gaining Trust**

In general, what could a dental office do to gain your trust for using their service?

(Please be as specific as possible)

| **Dental Attributes** |
| --- |

**Q6. Importance of Dental Office Attributes**

When thinking about your current dental office, how important are each of the following statements?

(Select one for each statement)

1. Extremely Important
2. Very Important
3. Somewhat Important
4. Not Very Important
5. Not at all Important

Personalized Communication

1. My dental team cares about me as a person and not just as a patient
2. My dental team’s way of communicating makes me feel like they relate to me in a personal way
3. My dental team’s home care recommendations are personalized for me

Immediate Gratification

1. My dental team offers immediate and convenient solutions
2. My dental team offers oral care products from the practice at a professional discount
3. My dental team recommends top of the line clinically proven products to keep me from spending extra personal time figuring out what is best for my personal health
4. My dental team cares about my time

Positive Health Outcome Linked to the Practice

1. My dental team has improved my oral health
2. My dental team has improved my overall quality of life

Other

1. My dental team has been in practice for a long time
2. My dental team is gentle
3. My dental team uses cutting edge treatments/technology
4. My dental team is the lowest cost

Teledentistry

1. My dental team uses email communication
2. My dental team uses social media/Facebook/etc. communication

**Q7. Likelihood of Behaviors**

How likely are you to do the following?

(Select one for each statement)

1. 5 – Definitely
2. 4
3. 3
4. 2
5. 1 – Definitely Not
6. I would recommend my dental office to my family and friends
7. I would trust and follow the treatment recommendations of my dental office
8. I would value the visit and keep my appointments
9. I would keep a long-lasting relationship with my dental office

| **Dental Recommendations and Purchases** |
| --- |

**Q8. Expectations of Dental Recommendations**

What do you, yourself, expect a dental office to recommend to their patients?

(Select all that apply)

1. Generic oral care products
2. Specific brand(s) based on my oral care conditions or to treat my specific issues
3. Only products/brand backed by clinical research to treat specific issues
4. Other (specify)
5. None of the above

**Q9. Option to Purchase Power Toothbrush from Dental Office**

Would you like the option to purchase a power toothbrush from a dental office?

(Select one)

1. Yes
2. No

**Q9a. Why Purchase Power Toothbrush from Dental Office**

Why would you like the option to purchase a power toothbrush from a dental office?

(Select all that apply)

1. I don’t want to make extra shopping trips
2. I want to make sure I purchase what my dental team prescribes
3. My dental office provides me with their professional discount
4. Other (specify)

| **Patients’ Understanding Towards Sampling** |
| --- |

**Q10. Importance of Samples**

What samples are most important to you after your dental visit?

(Select one for each)

1. 5 – Most Important
2. 4
3. 3
4. 2
5. 1 – Least Important

Toothbrush

Floss

Toothpaste

Mouthwash

Whitening strips

**Q11. Power Toothbrush Refill vs. Manual Brush Sample**

Would you prefer a power toothbrush refill over a manual brush sample?

(Select one)

1. Yes
2. No

**Q12. Added Value Samples**

What would add value to your dental visit?

(Select all that apply)

1. Provide samples
2. Provide a goody bag with samples and educational information
3. Provide samples specifically recommended for you
4. Dental office’s name imprinted on a toothbrush
5. Other (specify)
6. None of the above
